# Insights from focus groups with trans and gender-diverse people with endometriosis: stories you tell, stories you don’t

**DOI:** 10.1080/26410397.2025.2562682

**Published:** 2025-09-17

**Authors:** Maddalena Giacomozzi, Jasmine Brazelton, Khushi Jeswani, Donna Ruumpol, Petra Verdonk, Annemiek Nap

**Affiliations:** a Researcher, Activist, Medical Doctor, Radboud University Medical Hospital, Nijmegen, Netherlands; President and Co-founder, Treat it Queer Foundation, Nijmegen, Netherlands.; b Researcher and Activist, Radboud Universiteit, Nijmegen, Netherlands; c Researcher and Activist, Treat it Queer Foundation, Nijmegen, Netherlands; d Researcher, Radboud Universiteit, Nijmegen, Netherlands; e Associated Professor and Activist, Amsterdam University, Amsterdam, Netherlands; f Professor, Gynaecologist, Radboud University Medical Hospital, Nijmegen, Netherlands

**Keywords:** endometriosis, transgender, health disparity, focus groups, LGBT health

## Abstract

Endometriosis is a chronic condition characterised by cyclic pain symptoms that often significantly affect health-related quality of life. Predominantly framed as a “woman's condition”, current research overlooks the experiences of transgender and gender-diverse (TGD) individuals with endometriosis. This jeopardises the right to health for a community that faces historically rooted social and health disparities. This study aims to explore the embodied relationship between gender and endometriosis symptoms among TGD people living with endometriosis. A secondary objective is to examine the accessibility and competence of healthcare systems in addressing the needs of this community. The methodology included two focus group discussions conducted across four focus groups (4 × 2). Fourteen participants representing diverse gender identities, various stages of endometriosis and ages, were recruited online from nine countries across three continents, forming a heterogeneous group. Reflexive thematic analysis identified 15 codes and 7 clusters. The results were organised into themes, following the four embodiment epidemiological notions. Participants reported their gender self-perception and endometriosis symptoms to be interrelated and mutually influential. Feelings of disconnection and alienation were prevalent, particularly during life events such as menarche. Due to mistrust and experiences of discrimination, many TGD individuals withhold critical information during medical consultations. TGD people with endometriosis have unique health needs, e.g. how to combine gender-affirming and endometriosis care, and they are often concerned about future employability. Overall, the study underscores the urgent need to improve healthcare for TGD individuals with endometriosis as a matter of health justice.

## Plain language summary

Endometriosis is a chronic health condition that causes painful menstruation. So far, it has mostly been studied as a condition that only affects women. Because of this, there is not much known about the experiences of transgender and gender-diverse (TGD) people with endometriosis. This lack of information makes it harder for TGD people to get the care they need. This study explored how gender identity and endometriosis symptoms are connected for TGD people, and how healthcare systems could be more responsive to their needs. Fourteen participants from nine countries took part in group discussions. The researchers analysed the group discussion transcripts and identified common themes among participants. For example, they found that gender identity and endometriosis symptoms strongly influence each other. In fact, when menstruating, many participants felt disconnected or alienated from their body. Specific life events, like the first time they had their period, were particularly challenging for most participants. Participants experienced discrimination in the healthcare system. This led to mistrust in healthcare providers, which meant they hid important health information from doctors, such as the fact that they were taking testosterone. Participants also worried about future employment and long-term consequences of endometriosis. The study concludes that TGD people with endometriosis face serious challenges in getting appropriate care for endometriosis. It is very important to quickly make improvements in healthcare systems to ensure fair, respectful, and appropriate treatment for TGD people.

## Résumé

L’endométriose est une maladie chronique caractérisée par des symptômes douloureux cycliques qui affectent souvent significativement la qualité de vie. Considérant qu’il s’agit principalement d’une maladie « féminine », la recherche actuelle néglige l’expérience des personnes transgenres et de genre divers atteintes d’endométriose. Cela compromet le droit à la santé d’une communauté confrontée à des disparités sociales et sanitaires profondément enracinées dans l’histoire. Cette étude vise à explorer la relation matérialisée entre le genre et les symptômes de l’endométriose parmi les personnes transgenres et de genre divers souffrant de cette affection. Un objectif secondaire est d’examiner l’accessibilité et la compétence des systèmes de santé pour répondre aux besoins de cette communauté. La méthodologie comportait deux discussions réalisées avec quatre groupes d’intérêt (2 × 4). Quatorze participants représentant diverses identités de genre, plusieurs stades d’endométriose et différents âges ont été recrutés en ligne dans neuf pays sur trois continents, formant un groupe hétérogène. Une analyse thématique réflexive a identifié quinze codes et sept groupes. Les résultats ont été organisés par thèmes, suivant les quatre notions épidémiologiques de matérialisation. Les participants ont indiqué que la perception de leur genre et les symptômes de l’endométriose étaient liés et s’influençaient mutuellement. Les sentiments de déconnexion et d’aliénation étaient prédominants, en particulier lors d’événements de la vie comme les premières règles. En raison de la méfiance et des discriminations subies, beaucoup de ces personnes cachent des informations essentielles pendant les consultations médicales. Les personnes transgenres et de genre divers atteintes d’endométriose ont des besoins de santé particuliers, par exemple comment combiner l’affirmation du genre et les soins liés à l’endométriose, et elles sont souvent préoccupées par leur future employabilité. Dans l’ensemble, l’étude souligne combien il est urgent d’améliorer les soins de santé pour les personnes transgenres et de genre divers atteintes d’endométriose dans un souci de justice sanitaire.

## Resumen

La endometriosis es una enfermedad crónica caracterizada por síntomas de dolor cíclico que a menudo afectan de manera significativa la calidad de vida relacionada con la salud. Las investigaciones actuales, que predominantemente la definen como una “afección de la mujer”, hacen caso omiso de las experiencias de las personas transgénero y de género diverso (TGD) con endometriosis. Esto pone en peligro el derecho a la salud para una comunidad que enfrenta disparidades sociales y de salud arraigadas históricamente. Este estudio pretende explorar la relación entre género y los síntomas de endometriosis entre las personas TGD que viven con endometriosis. Un objetivo secundario es examinar la accesibilidad y competencias de los sistemas de salud para atender las necesidades de esta comunidad. La metodología consistió en dos discusiones en grupos focales realizadas con cuatro grupos focales (4 × 2). Se reclutaron en línea catorce participantes que representaban diversas identidades de género, y diversas etapas de la endometriosis y edades, provenientes de nueve países en tres continentes, que formaron un grupo heterogéneo. El análisis temático reflexivo identificó quince códigos y siete clústeres. Los resultados fueron organizados por temas, siguiendo las cuatro nociones epidemiológicas de encarnación. Las participantes informaron que su autopercepción de su género y los síntomas de endometriosis estaban interrelacionados y eran mutuamente influyentes. Los sentimientos de desconexión y alienación eran prevalentes, en particular durante vivencias como la menarquia. Debido a la desconfianza y experiencias de discriminación, muchas personas TGD ocultan información fundamental durante las consultas médicas. Las personas TGD con endometriosis tienen necesidades de salud únicas, tales como cómo combinar la atención de afirmación de género y el tratamiento de la endometriosis, y a menudo se preocupan por su futura empleabilidad. En general, el estudio subraya la necesidad urgente de mejorar los servicios de salud para las personas TGD con endometriosis como un asunto de justicia social para la salud.

## Introduction

### Background

Endometriosis is a condition defined by the presence of endometrium-like tissue outside of the uterine lining.^1^ Its leading symptoms are cyclic and chronic pelvic pain, both of which have a profound impact on individuals’ lived experiences and health-related quality of life.^2^ Additional endometriosis symptoms include subfertility, dyschezia (painful defecation), dysuria (painful urination), and sexual problems/dysfunctions.^3^ Sexual problems/dysfunction include dyspareunia (painful intercourse) and painful orgasm. Symptoms typically present throughout one’s reproductive life phase, meaning between early teen years until menopausal onset.^1^ However, recent studies show that they can persist past menopause.^4,5^ They can affect several life domains, including careers and intimate relationships, resulting in an overall decrease in health-related quality of life.^6^ The effects of this condition affect one’s life course from reduced employment opportunities to jeopardised family planning.^7^ Due to its progressive character and extensive consequences, endometriosis often requires long, multidisciplinary and expensive treatment plans tailored to one’s life stage which are not easy to access.^1^ Despite its immense impact on health-related quality of life, this condition is frequently under- and misdiagnosed with diagnostic delays of several years.^8–11^ Moreover, similarly to many chronic pain conditions, endometriosis is frequently misunderstood and stigmatised by healthcare providers (HCPs) and the overall social environment of those living with it.^2^

The prevalent gendering of menstrual pain as a “woman’s condition” implies that endometriosis in the dominant discourse is seen, narrated and taught to be a condition affecting only cisgender women.^12^ Since endometriosis affects individuals with what are typically considered “female” reproductive anatomy, discourse around and treatment of endometriosis focuses on femininity and “female” fertility.^13–15^ Such discourse excludes individuals with endometriosis who have bodies and identities which fall outside of normative conceptions of cisgender (white) womanhood.^13,16^ Thus most endometriosis research excludes transgender and gender-diverse (TGD) people, who are individuals whose gender identity is different from the gender assigned to them at birth. This study focuses on people assigned-female-at-birth (AFAB) who identify with genders other than “female”. Some examples of other gender identities are “transgender male”, “nonbinary”, “genderfluid” and “genderqueer”. Endometriosis has been diagnosed across a range of genders and anatomies.^15^ Limited evidence suggests that endometriosis is more prevalent among TGD individuals compared to the AFAB population.^17,18^

TGD people face structural discrimination and systematically encounter great barriers to access healthcare, and gynaecological healthcare services in particular.^19–21^ They report overall worse health outcomes than cisgender people, with some studies indicating that nonbinary and genderfluid individuals report poorer health than binary transgender individuals.^22–24^ Gynaecological services are especially hard to access due to a combination of factors such as lack of education and negative attitudes of HCPs towards TGD persons, and gynaecological physical examinations may possibly trigger gender dysphoria. TGD people have distinct and unique health needs compared to the cisgender population. This is partially attributable to the fact that some TGD people need gender-affirming care (GAC), and that may interfere with other conditions or treatments. GAC entails a wide spectrum of possible medical and non-medical strategies to affirm one’s gender.^25^ The most common masculinising medical GAC options are hormonal (e.g. use of exogenous testosterone) and surgical (e.g. removal of reproductive organs such as hysterectomies and oophorectomies).^25^ Endometriosis places a significant burden on TGD individuals, not only because it disproportionately affects this community but also due to the limited resources available to meet their health needs.^16,26^ The underlying mechanisms in which GAC and endometriosis progression and treatment may interact remain largely unexplored.^12,26^ Exogenous testosterone is found to suppress the menstrual cycle insufficiently in many individuals during long-term use (>1 year).^27^ In such cases, it is hypothesised that endometriosis would also not be adequately treated.^12,28^ The pervasive lack of studies on endometriosis among TGD people prevents a comprehensive and rights-informed understanding of how this community experiences not only endometriosis symptoms and care but also their relationship to their genders and menstrual cycles. This neglect constitutes a violation of the right to health as defined by the World Health Organization (WHO), which requires health systems to ensure availability, accessibility, acceptability, and quality (AAAQ) of care for all.^29^ TGD individuals face profound barriers in all four dimensions: *availability* is limited due to the scarcity of competent, gender-affirming gynaecological services; *accessibility* is undermined by systemic discrimination and structural barriers to adequate care; *acceptability* is compromised by cisnormative practices that exclude gender-diverse experiences from health policy; and *quality* is lacking due to insufficient research and training on TGD-specific needs, resulting in ineffective and inadequate care. This systemic failure to uphold the core components of the right to health perpetuates health inequities and reinforces the marginalisation of TGD individuals. It is thus crucial to explore how TGD people with endometriosis experience their bodies, cycles, and symptoms in relation to themselves and their environments to develop healthcare systems that are not only inclusive and equitable but also aligned with fundamental human rights principles. Identifying challenges as well as resilience strategies within and beyond the formal healthcare system is necessary to address their health needs in a manner consistent with the ethical and legal imperative to fulfil the right to health and promote health justice.

### Aim of study

The primary objective is to gain insights into how TGD people living with endometriosis experience the embodied relationship between their gender and endometriosis symptoms. The secondary objective is to investigate the accessibility and competence of the healthcare system for TGD persons with endometriosis. This study aims to recentre the marginalised voices and stories of TGD people with endometriosis through focus group discussions to ultimately improve the healthcare system and narrow health disparity.

### Theoretical framework

This study draws from Nancy Krieger’s scholarship on embodiment.^30–32^ It is designed on the premise that humans are simultaneously social beings and biological organisms whose lives literally embody “the dynamic social, material, and ecological contexts into which they are born, develop, interact, and endeavour to live meaningful lives”.^30^ These processes develop and express throughout the life course.^31,32^ This study focuses on – but is not limited to – the period of one’s life most affected by endometriosis, i.e. the reproductive phase. The study is rooted in the tenet that bodies are framed as more than mere physical entities that experience health or illness dichotomously, and their investigation is taken as an opportunity to understand embodiment pathways that may be caused by, or lead to, disparities including health disparities.^31^ Bodies are thus understood as potential epistemological mines for phenomenological insights into the “historically contingent, spatial, temporal, and multilevel processes [that] become embodied and generate population patterns of health, disease, and wellbeing, including social inequalities in health”.^30^ Understanding collective and individual experiences as being embodied throughout historical processes, as well as singular life courses, implies appreciating that bodies tell stories. According to Krieger,^30^ bodies can tell different stories that can be divided into (1) stories about the conditions of our existence; (2) stories that match people’s stated accounts; and (3) stories that people cannot or will not tell because they are unable to, or are forbidden, or they choose not to tell them. Embodiment as an epistemological notion can be understood in a fourfold manner as:

- a construct, process, and reality, contingent upon bodily existence;
- a multilevel phenomenon, integrating soma, psyche, and society, within historical and ecological context, and hence an antonym to disembodied genes, minds, and behaviours;
- a clue to life histories, hidden and revealed;
- a reminder of entangled consequences of diverse forms of social inequality.^30^

Embodiment as an epistemological notion is helpful in investigating and addressing heterosexism and gender binarism in health equity research through an ecosocial analysis.^31^ Other authors have successfully applied the embodiment framework to explain health disparities experienced by TGD people. For example, Duffy et al^33^ examined the prevalence of self-injurious thoughts and behaviours in transgender individuals with eating disorders compared to cisgender individuals with eating disorders and transgender individuals without eating disorders. Embodiment is helpful also in understanding how bodily-reflexive practices are central for transmasculine people for doing masculinity.^34^ In fact, it offers a framework to more comprehensively understand how bodily experiences shape and redefine masculinities beyond medicalised practices and hegemonic masculine norms.^34^

This theoretical framework was chosen to explore the stories of an otherwise systemically marginalised and silenced community such as that of TGD people with endometriosis in their ecosocial context throughout their life courses. As such, this study was conducted based on the foundation of rejecting biological essentialism and definitions of social normality or deviance based on biological essentialism. The foci of the current study are *emergent embodied phenotypes* as defined by Krieger.^32^ These bodily phenomena are thus studied as the biological expressions of unjust systems, and in this case as adverse embodied consequences of two main power axes, structural heterosexism and gender binarism.

## Methods

Qualitative research methods were selected to answer the research questions. Because of the pervasive lack of pre-existing literature on this topic, an explorative study was conducted using focus groups among gender-diverse people living with endometriosis.^12^ Qualitative methodology was selected to answer the research questions while optimising the wide geographical spread and relatively low number of TGD people living with endometriosis. The Standards for Reporting Qualitative Research (SRQR) guidelines guided the reporting of the current study.^35^

### Qualitative approach and research paradigm

**Figure 1.**
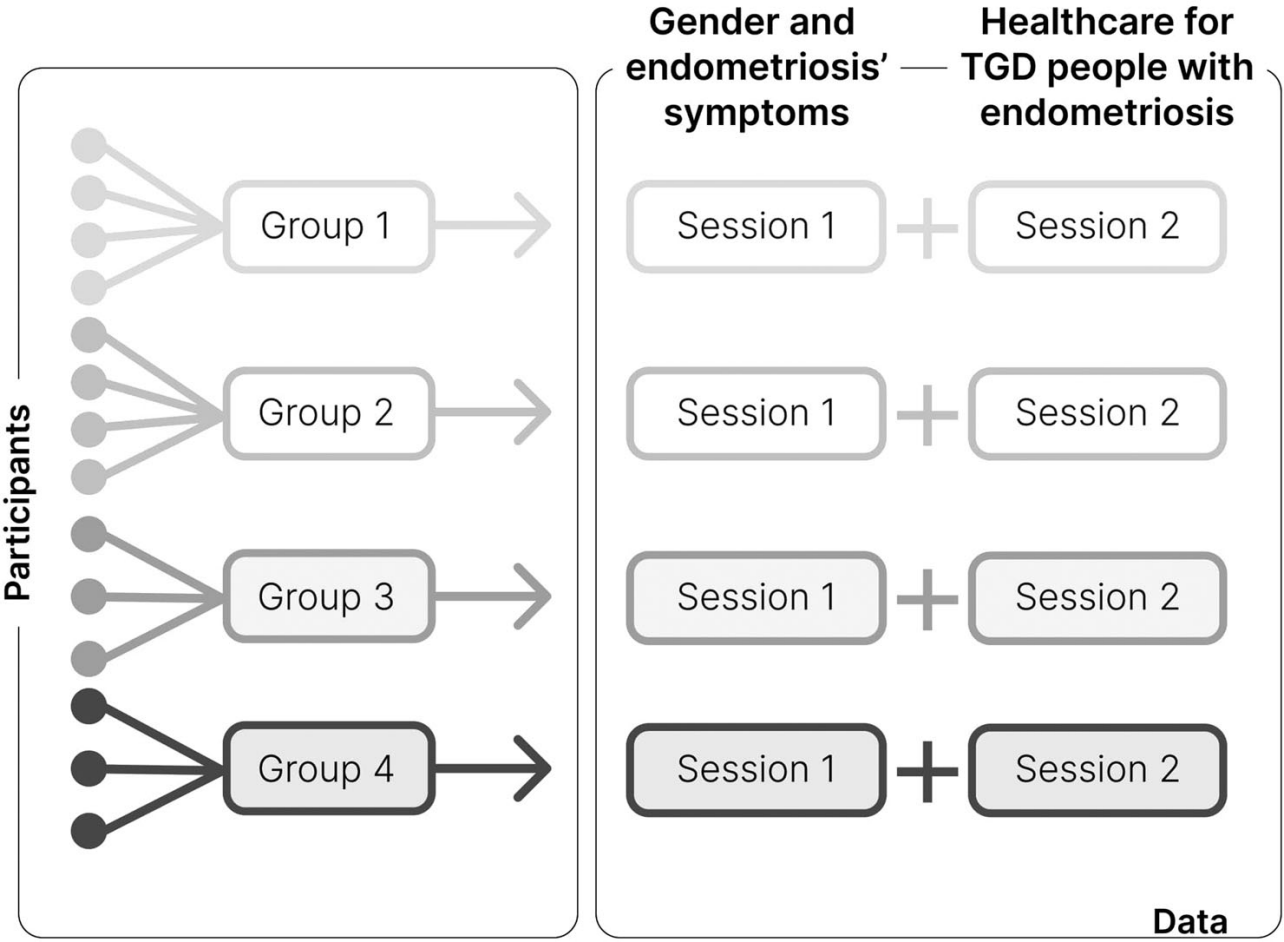
Focus group methodology

The research paradigm of this study draws on embodiment theory based on Krieger’s scholarship as outlined in the Introduction. The qualitative approach of this study was based on focus group methodology (Figure 1). Four online focus groups were facilitated, each of them for two sessions, which resulted in a total of eight sessions. Each session was scheduled for 1.5 hours. Thus each group was interviewed twice for about 1.5 hours, so in total approximately for 3 hours. Groups were formed based on participants’ availability for specific timeslots. Demographics were not accounted for in the grouping process. Each group included between three and four people, for a total of 14 participants, to enable a smooth conversation online and to allow everyone enough time to elaborate on their answers. Participants were not allowed to switch groups in between sessions. During each session, a facilitator and a notetaker were present to moderate the discussion. The facilitator was the same in the first and second sessions, while the notetaker could change based on the research team’s availability.

### Research team characteristics and reflexivity

The research team was TGD-led. The first author and facilitator of all focus group sessions [MG], identified as nonbinary transmasculine and had previous experience with facilitating focus groups and with qualitative research methods. The research team consisted of half TGD people and half cisgender women. One TGD person [JB] with endometriosis was part of the research team and had personal experience with coping mechanisms for living with endometriosis and chronic pain. All people in the team had a strong affinity with the lesbian, gay, bisexual, and transgender (LGBT+) communities, either because they were themselves community members, or because they had close personal connections with community members, e.g. their children. Most of them had previous experience in working in LGBT+ health research and/or LGBT+ advocacy. The subjectivities of the researchers favoured the application of what McDonald^36^ describes as a queer reflexivity lens on the research question and data analysis. That perspective resulted in a reflexive and circular questioning of the categories that were used to identify people while recognising the shifting nature of the identities of both the researchers and the participants – not only during their life courses but also during the research process.^36^ Similarly, the nonbinary embodiments of the facilitator [MG] and of other researchers [JB, KJ] facilitated an expansive understanding of embodiment and guided the research design as well as the implementation of the study.^37^ This proximity to the TGD community specifically, and LGBT+ at large, implied that the research team was well familiarised with queer culture and queer jargon prior to the study. The team varied in terms of age and seniority in research from bachelor intern to professor. Diversity in age and life experience facilitated the interpretation of data collected with participants of different ages, especially juxtaposing data from teenagers and middle-aged individuals. Various disciplinary backgrounds were represented, including global public healh, psychology, sociology, anthropology, neuroscience, biomedical sciences and medicine – specifically obstetrics and gynaecology – and sexology. Half of the team had a background in advocacy and activism besides academia. All team members were based in the Netherlands during the implementation phase. However, half of the members originated from another country. The team was predominantly white.

### Context

The focus groups were conducted online to enable accessibility of people living in different geographical areas, including rural and remote settings. The digital context was expected to facilitate the creation of enriched data from individuals across intersectional backgrounds, particularly by facilitating the access of people with chronic illness experiencing barriers for mobility. The discussions were conducted in English.

### Sampling strategy

Recruitment took place online through a call spread through social media groups and newsletters. LGBT+ groups as well as groups of and for people living with endometriosis engaged to share the call. It was mostly shared on channels of organisations based in Global North countries. The research group did not approach patients in their hospital. Reflexive thematic analysis was conducted throughout the research process, with codes generated and themes identified following the approaches of Braun and Clarke^38^ and Naeem et al.^39^ Recruitment was discontinued when no new information or themes emerged from the data, i.e. when information redundancy was observed by the researchers during data collection, indicating that further sampling was unlikely to yield additional insights. Inclusion criteria included: identifying as transgender and/or gender diverse while being AFAB, being at least 18 years old, speaking sufficient English to partake in group discussions, and having been diagnosed with one of the currently recognised diagnostic methods for endometriosis in the ESHRE guidelines 2022 (i.e. transvaginal ultrasound, Magnetic Resonance Imaging (MRI), or diagnostic laparoscopy).^1^ Endometriosis diagnosis was self-reported. All types and stages of endometriosis were included. All self-identified TGD people could participate regardless of whether they had a diagnosis of gender incongruence/dysphoria or whether they were accessing GAC. Exclusion criteria were being assigned-male-at-birth or younger than 18 years, identifying as a cisgender woman, insufficient command of the English language, suspected yet not confirmed endometriosis, and self-diagnosis of endometriosis. Comorbidities were self-reported in the Qualtrics survey and were not a reason for exclusion.

### Ethical considerations

The present study included human participants. However, due to the non-invasive nature of the study, the ethics committee of Radboud University Medical Center, METC Oost Nederland, exempted it from ethics approval (1 March 2024). All participants gave written informed consent prior to enrolment.

### Data collection methods

Focus group discussions were conducted between 23rd March 2024 and 4th May 2024. The interval between the first and second sessions varied per group based on their availability, and it ranged from one to three weeks. The discussion guide (Annex A) could be modified throughout the process to follow an organic evolution of the group discussion. The triangulation of data was not conducted. The loss of follow-up by participants between first and second was reported and the reason for loss of follow-up investigated. The reason for the drop-out was poor internet connection and miscalculation of time zones for the scheduling of the call. Focus groups were chosen as the primary method to facilitate comprehensive, insightful, and interactive discussions that capture the collective experiences and diverse perspectives of participants. This method was considered particularly appropriate for exploring complex and under-researched topics, like the lived experiences of endometriosis, and for centring the voices of historically marginalised communities, such as TGD individuals.

### Data collection instruments and technologies

Participants provided their demographic data through an online survey through Qualtrics prior to the focus group discussions. In this survey, they self-reported on how endometriosis was diagnosed and on comorbidities. Written discussion guides steered the conversations, which were held online and recorded. A second sound recording device was used next to the laptop to provide a back-up recording of the same focus group discussion. Live transcription was available for all participants to facilitate interaction, especially for non-native speakers and neurodivergent participants.

### Units of study

Demographics, including participants’ gender and medical background, were self-reported. No medical file nor documentation were required. Discussion files were saved both in audio-only and video format. Live transcripts were written as notes.

### Data processing and analysis

The focus group sessions were recorded with participants’ knowledge and consent. Audio and video files were stored in the secured drive of the leading research centre with restricted access according to the local Data Management Plan (DMP). They were only visible to the researchers. No other third party could access or store this data. Anonymisation was conducted on all data prior to publication.

### Data analysis

Live transcripts and video files were analysed. These were transcribed verbatim. Tables exported from Qualtrics were manually analysed to deduct the demographic data. Focus groups and audio-only files were coded manually primarily by one researcher and checked by two other team members. In the event of a disagreement, a third team member was consulted, and discussions were conducted until consensus was reached. Codes were subsequently clustered. Braun and Clarke^38^ and Naeem et al^39^ guided the reflexive thematic analysis of the data. This method included six steps, namely:

1. data familiarisation and writing familiarisation notes;
2. systematic data coding;
3. generating initial themes from coded and collated data;
4. developing and reviewing themes;
5. refining, defining, and naming themes;
6. writing the report.

### Techniques to enhance trustworthiness

The research questions as well as the discussion guide and recruitment call were developed in consultation with TGD people with endometriosis. Several community member checks were conducted at different stages of the research designing and implementing phase. The strong involvement of the research team with the target community further enhanced the trustworthiness of the study and guaranteed an efficient recruiting strategy. No audit trials were required.

## Results

### General findings

Forty-six individuals (aged 19–49 years) responded to the call for participants. One person was excluded because endometriosis had not been confirmed in their case. Participants were recruited on a rolling basis according to their availability for online meetings. Focus groups were conducted until no new themes emerged and sufficient heterogeneity in group composition was achieved. In total, 14 participants took part in the discussions (*N* = 14), which were held across four focus groups (see Table 1). Through reflexive thematic analysis, information redundancy was reached after six discussions across three groups. To ensure no themes had been overlooked and to confirm that participants had no additional contributions, two additional discussions with a fourth group were conducted. Two participants dropped out between the first and second sessions. Demographics of the participants are described in Table 1.

**Table 1.** Demographic characteristics of participants (n=14)

Individual-level variable	N
**Gender**	
* Nonbinary*	5
* Nonbinary transmasculine*	2
* Nonbinary transman*	1
* Nonbinary trans, genderqueer*	1
* Nonbinary, butch, transmasc*	1
* Gender-nonconforming*	1
* Ambiguous*	1
* Queer*	1
* Agender/demigender*	1
**Pronouns**	
* They/them*	6
* She/they*	3
* They/he/she*	1
* They/she/he*	1
* He/him*	1
* They/he*	1
* Any/all*	1
**Race**	
* White*	13
* Mixed*	1
**Country where health care was sought**	
* The Netherlands*	3
* Germany*	3
* Italy*	3
* United States of America (USA)*	2
* Greece*	1
* Austria*	1
* United Kingdom (UK)*	1
* Australia*	1
* Canada*	1
* of which more than one country*	2
**Endometriosis symptoms**	
* Dysmenorrhea*	10
* Dysuria*	4
* Dyschezia*	5
* Chronic pelvic pain*	12
* Other*	3
**Endometriosis diagnosis**	
* Ultrasound*	4
* MRI*	2
* Diagnostic laparoscopy*	13
**Hormonal medications**	
* COCs*	0
* Progestins-only*	8
* GnRH analogues*	0
* GnRH-antagonists*	0
* Danazol*	1
* Testosterone*	4
**Current painkiller use**	
* Yes*	10
* No*	4
**Previous surgeries**	
* Diagnostic laparoscopy^a^*	13
* Other laparoscopic abdominal surgeries*^b^	9
* Gender-affirming mastectomies*	2
**Comorbidities**	
* Somatic*	5
* Psychological*	2
* Mixed*	2
* Use of medication for comorbidities*	8

^a^In case endometriosis was detected, the same surgery included ablation and/or excision of endometriosis lesions.

^b^These included five hysterectomies, two bilateral oophorectomies, one adhesiolysis and one tubal ligation.

### Reflexive thematic analysis

#### General

Data analysis led to the identification of 15 codes. The codes were grouped into seven clusters. These were: gender and the cycle; pain; menarche; life stories; barriers; diagnosis; treatment. These clusters were subsequently reorganised in four themes according to the four epidemiological notions of “Embodiment: a conceptual glossary for epidemiology” and presented below in this arrangement (Table 2).

**Table 2.** Reflexive thematic analysis following embodiment epidemiological notions

Embodiment epidemiological notion	Theme	Subtheme
A construct, process, and reality, contingent upon bodily existence	*Constructing an embodied understanding of gender and the menstrual cycle as reciprocal processes*	NA
A multilevel phenomenon, integrating soma, psyche, and society, within historical and ecological context, and hence an antonym to disembodied genes, minds, and behaviours	*Contextualising cyclic pain as a multilevel phenomenon integrating somatic, psychological and social contributing factors*	NA
A clue to life histories, hidden and revealed	*Bodies (un)telling life histories*	*Crossroads between endometriosis care and GAC; Menarche and (failing to) enter “womanhood”; Reproductive health; Biographical disruptions and concerns for the future*
A reminder of entangled consequences of diverse forms of social inequality	*Accessing endometriosis care while navigating the consequences of social inequalities*	*Barriers to diagnosis; Barriers to (effective) treatment*

#### Constructing an embodied understanding of gender and the menstrual cycle as reciprocal processes

The focus group discussions made clear that gender is perceived by TGD people as a process with neither a defined trajectory nor a definitive ending point. For most participants, understanding gender as relational and contextual meant seeing its evolution as inevitable and intrinsic to its ontological characteristics, whether they described it as “fluid”, “layered” or otherwise. Once operationalised into different facets, some nuanced their reflections about “gender”, stressing that gender expression could be differently susceptible to change than gender identity. Further, gender attribution played a major role in their gender self-perception, especially for people who medically transitioned later in life. Age played a significant role in one’s relationship with gender, and many described how this relationship transformed throughout different life phases depending, for example, on their partners, family planning and career choices. Accepting gender fluidity, versatility, and unpredictability was a landmark in many participants’ journeys with chronic illness and gender affirmation.

“*There is a question in me that may always be present about what my gender is. [My gender] might never be stationary it might always be in flux. I started transitioning as a middle-aged person in my mid-forties, and so that's going to be really different from perhaps someone that hasn't lived an entire life as a particular gender*.” (B., nonbinary transman in his 40s, USA)

Similarly, according to participants, it was self-explanatory that the menstrual cycle is a more-or-less monthly process through which one’s gender feelings, bodily perceptions, and physical experiences significantly change.

“*I did notice how my own self-perception changed when the hormones also switched*.” (A., queer in their 20s from Germany)

Cyclic pain played a major role in defining the cycle. In fact, all participants experienced it for consistent amounts of time (usually years if not decades), and 10 people were still suffering from it regularly. When asked how pain made them feel in their bodies, this answer was paradigmatic:

“*[Menstruating] was a living hell, I felt disconnected from my body during the menstrual phase. Really terrible image of myself. I felt miserable but I didn’t know why, I didn’t have the words to describe it*.” (N., nonbinary person in their 20s from Italy)

Meanwhile, the comprehension of the menstrual cycle as a gendered phenomenon influenced the experiencing of the cycle. When asked “how do you relate to your menstrual cycle?” many participants talked about how social gendering of menstruation affected them.

“*I had always thought that “oh a period or menstruation is like a woman's thing”. I think I felt more feminine [when menstruating] but in a bad sense, like a femininity that was imposed on me. I think that kind of info influenced how I perceived myself in those days as well*.” (H., nonbinary person in his 20s from Greece)

The consequences of gendering menstruation for TGD people are further explained by the claim by D., a nonbinary person in their 20s from Canada:

“*If periods were not just a gendered thing, I would probably not feel the same level of dysphoria, but that is unfortunately not the society we live in*.”

Contrastingly, people who had taken measures to effectively suppress their cycle (e.g. hysterectomy and bilateral oophorectomy) reported diametrically opposite feelings:

“*I feel so much more solid without all the hormonal changes all the time*.” (S., nonbinary person in their 40s from USA)

For several people, understanding their changes in (either endogenous or exogenous) oestrogens and testosterone were turning points in their “gender journey”. Their gender perceptions shifted after realising that their psychological status was improving with higher levels of testosterone and deteriorating with the elevation of oestrogens.

“*I also used to only have like three days so I would feel like really good about myself and my body […] then I realized that the days I usually felt really good about myself are the days with the most testosterone in my body. I felt like the best. […] These were the days I felt like myself*.” (P., nonbinary trans genderqueer in their 30s from Germany)

“*I was going through early menopause, and my doctor gave me hormones that included testosterone. That changed my feelings about myself so dramatically and I knew that testosterone was the medicine that I needed. My next step was to stop all female hormones and to get on male hormones completely. And I felt so much better. […] As far as my gender goes, I didn’t realize how trapped I felt in my gender until I no longer menstruate, and that sense of freedom was incredible and that is when my sense of gender begun to seriously fluctuate*.” (B., nonbinary transman in his 40s, USA)

The interaction between gender and the menstrual cycle is typically not viewed as a one-way influence, where one variable affects the other in isolation. Instead, it is understood as a reciprocal and contingent relationship, involving multiple dimensions of an individual's life experience. While the association became clearer, the causality did not. When asked how they related to gender throughout their cycle, a person gave this example of how menstruating affected them:

“*The feeling of not having control and not being able to be active [while menstruating] was hard on my mental health. I barely can leave the house, I am not really doing any exercise, not even able to go to the office very much, so the feeling of not being able to do the things does not make me feel like myself. It was also very dysphoric. Often something I do to help dysphoria is doing sports, but when you sit in your bed for like a week it doesn’t give you many outlets for trying to find gender euphoria*.” (R., transmasculine non-binary person in their 20s from UK and Austria)

#### Contextualising cyclic pain as a multilevel phenomenon integrating somatic, psychological, and social contributing factors

TGD people with endometriosis reported classical endometriosis-related pain, including dysmenorrhea, dysuria, and dyschezia. The majority of them (*n* = 13) indicated having chronic pelvic pain. Participants referred to “chest” and “top” instead of “breasts”. They described non-typical pain in the chest area as one of the most impactful symptoms, but HCPs usually did not ask about it and often failed to recognise it as iatrogenic or endometriosis related. This was often described as an intertwinement of chest tenderness and top dysphoria, and occasionally as a side effect of combined oral contraceptives (COCs) which were discontinued by all participants who tried them.

They further elucidated how pain triggered a negative spiral. Pain made them feel differently in their bodies, and the gendered layering of menstrual pain contributed to gender dysphoria. In fact, participants reported that they “become more aware” and “were forced to connect” to certain body parts that in turn caused feelings of “dissociation” and “alienation”.

“*Pain is always a message, the body is telling you ‘Please, take care of me’, it’s a conversation. But during those experiences I dissociate a lot*.” (Z., a non-binary transmasculine butch person in his 20s from Italy)

“*[Menstruating] really made me not want to be a woman, you know? ‘Cause if that's what it is. Why would I want it, you know? I definitely felt different in my body when I was in pain. I kind of was dissociating. Dissociating and being like, okay. Like it was just like something like you have to get through. Because you had no other choice*.” (A., queer person in their 20s from Germany)

Disconnection from their bodies was further related to gender dysphoria and aggravated thinking of being “stuck” and “trapped” in their bodies. The process was exacerbated by the impossibility to fulfil daily functions as prior to menstruation and by concerns about future consequences relating to career options and intimate relationships.

“*First days of menstruation … it’s like a truck. When it arrives it’s your whole life: gender, relationships, your carriers, field of study, everything*.” (E., agender/demigender person in his 20s from Italy)

The consequences of such disruptions were exacerbated by lack of adequate social support, as exemplified by this response:

“*As my endometriosis got worse, there are times when I can’t leave my bed for a week, and there is none to cook [for] me, to do errands for me, to do groceries*.” (K., nonbinary person in their 20s from Australia)

#### Bodies (un)telling life histories

All participants had lived with endometriosis for several years, sometimes decades, at the time of the study. Endometriosis symptoms impacted their lives in various ways depending on their life stage. Endometriosis consequences intersected with one’s gender journey in ways which were idiosyncratic to the individual. The onset of endometriosis symptoms was for all during adolescence, often parallel to the first feelings of gender dysphoria. Time of diagnosis varied between teenage years and late 30s. Steps in social and medical transition were taken at a different pace with some individuals starting GAC as minors, and others in their 40s. Four crucial points were identified in their life histories: menarche, family planning, biographical disruptions, and crossroads between endometriosis care and GAC.

### Crossroads between endometriosis care and GAC

Many participants above 30 years of age reported that specific endometriosis treatment contributed positively to their understanding of their gender, and at times it opened a gate to GAC.

“*Having my hysterectomy and no longer having a menstrual cycle really freed me to be able to address the gender dysphoria and begin transitioning*.” (B., nonbinary transman in his 40s, USA)

A gender-nonconforming person from the Netherlands explained that when they had their hysterectomy scheduled due to unbearable endometriosis-related pain, many cis-women showed concerns to them about their (future) inability to carry a pregnancy. They felt distance and dissonance from the cis-women reactions. The concerns spurred the participant’s reflection about what that surgery meant for them personally, and what it implied in the construction of the “female” gender through the “doing” of reproduction as a “woman”.

“*So many people asked me “omg you are so young, and you are going to lose your uterus, how is that for you? As a woman? […]” I’m fine with my body without a uterus, and I don’t feel like I’m losing my feminine side*.” (T., gender-nonconforming person in their 30s from the Netherlands)

### Menarche and (failing to) enter “womanhood”

Menarche was a critical point for everyone who participated in the discussions. Feelings around it were reflective of “unpreparedness”, “fear”, and “anxiety”. Its heavily gendered aspect, often accompanied by the onset of at-the-time-unexplainable pain, made it a challenging moment as well as a possibility to confront and reflect about themselves in relation to women and men. Even when explicitly asked, no participant recalled a story in which a father or male relative was actively engaged during this phase of their lives. Meanwhile, women, and especially mothers, are often described as “excited” for this moment. They framed it as a rite of passage into womanhood while dismissing and normalising the pain as something that is defining of womanhood. Female relatives were at the forefront of the gendered normalisation of pain, making claims such as: “pain for women is normal”; “to be a woman is to suffer”; and “all women in our family have this”. Consequently, participants were expected from a young age to “just get through it” and “suck it up, as they say, and pretend like it's not a big deal, as if I am not in tremendous pain”. This often led to feelings of “alienation”, “denial”, “confusion”, and “dissociation” in the people experiencing it.

“*I didn’t understand what was going on. I remember being so scared and I remember going to bed in pain, then waking up in pain. So, from the very first time, I remember I associated my period with being in pain, not being able to run around like a kid. […] When I told my mom she was like “oh, you're like becoming a woman” the classic things that people say. I remember I was like, but I don't wanna become a woman. I don’t want to do this*.” (H., nonbinary person in his 20s from Greece)

### Reproductive health

Endometriosis-related subfertility aroused various feelings among participants. In response to the question “What does endometriosis-related subfertility mean for you?”, many described the impossibility of carrying a pregnancy as a “relief”, since it freed them from the social expectations of compulsory motherhood. One participant from Germany underwent tubal ligation as GAC as their first abdominal surgery, and endometriosis was an accidental finding during that operation. Retrospectively, they had had symptoms since early puberty. On the other hand, two middle-aged participants were grieving being childless, and both would previously have liked to carry a pregnancy themselves. For one of them, learning that they would never become pregnant meant that they started questioning their gender since their body was not able to do “the only thing that it was supposed to do that I wanted”, and after their hysterectomy started using testosterone.

### Biographical disruptions and concerns for the future

Receiving the diagnosis of endometriosis was disruptive for most people with endometriosis and was perceived as “validating”, a “relief”, but also “concerning” and “terrifying” for what its long-term consequences could be. The main concerns related to future employability and financial security. Some participants were preoccupied about prospective intimate relationships due to sexual problems. Family planning and subfertility were background concerns for TGD people in early adulthood who experienced greater barriers to access assisted reproductive techniques (ARTs), especially when partnered with another AFAB person. Most countries of residence did not recognise endometriosis as a disability, and this resulted in insufficient welfare systems to support participants in partaking in study programmes or workforce to their full potential.

“*This is a scary part because I want to work in the university and I'm afraid that with my body … I could not stand a job […] I'm very scared that I cannot find a job that I like and for which I studied*.” (N., nonbinary person in their 20s from Italy)

#### Accessing endometriosis care while navigating the consequences of social inequalities

All TGD participants encountered barriers to access endometriosis case. These varied in relation to individual factors such as gender expression and stage of endometriosis, and contextual factors such as preparedness of single HCPs and organisation of the healthcare system.

### Barriers to diagnosis

Participants reported several diagnostic delays; however, this study did not collect range or average metrics about it. They attributed the delay to several contributing factors. Most participants’ help-seeking behaviour was one of them. At the time of symptoms onset, they usually depended on family members, especially mothers, to seek care. However, female family members appeared typically to be aggravating the gendered normalisation of pain (see above: *Menarche and (failing to) enter “womanhood”*). This meant that many young TGD people waited a few years before reporting their symptoms to professionals. HCPs further contributed to the delay in diagnosis by dismissing and undermining the reported symptoms, or by attributing them to a psychogenic cause. HCPs’ behaviour exacerbated the mistrust that TGD people have in the healthcare system, while leaving endometriosis untreated and thus progressing throughout the years.

“*For many many years I would go to doctors, I would go to gynecologists, and I would say hey I'm in a lot of pain and they would say “You're just anxious. You're just worried about exams at school”*.” (H., nonbinary person in his 20s from Greece)

#### Barriers to (effective) treatment

Nine participants were using hormonal medication to suppress the menstrual cycle, eight of which used progestins-only, and one danazol. Many tried COCs and all discontinued it due to side effects, often relating the choice to affective symptoms, gender dysphoria or chest/top tenderness. The participant who used danazol said about the medication:

“*Danazol has been literally life-changing. It’s also so gender affirming, and makes my pain very low, so I am able to function almost normally. And it has all the fun side-effects of testosterone*.” (J., nonbinary transmasc person in their 20s from the Netherlands)

Reflecting on “What makes the healthcare services you need hard to access?”, the most prevailing barrier to access adequate and competent care related to the gendering of gynaecological practices. This was more evident in the testimonies of people from Greece and Italy, and less obvious in North Europe and Canada.

“*If testosterone is on my medication list, I will have to come out to my gynecologist, and that really scares me. What if they discriminate me because of that?*” (Z., a non-binary transmasculine butch person in his 20s from Italy)

“*I have 5 or 6 healthcare providers […]. Most of my providers had a spot on their registration form for preferred name […]. Most of them are respectful of pronouns and a lot of them have worked with trans clients before so they know what to expect. They don’t try to push certain things on me, and they are just allowing me to be me*.” (D., a nonbinary person in their 20s from Canada)

Additional HCP-related barriers included the lack of education of HCPs about gender diversity and endometriosis and personal attitudes towards gender diversity. Lack of appropriate education was found to be pervasive in all the countries where participants accessed care. Acute settings were particularly problematic. One participant from the USA reported being misgendered by an HCP in the recovery room after their mastectomy while still on opioids. When they indicated their pronouns, the HCP required them to educate them on the spot about gender diversity. In countries with privatised healthcare systems, especially the USA, financial barriers posed a serious challenge for TGD people with endometriosis to access adequate care. Lack of financial security as a consequence of endometriosis and limited employment opportunities due to gender-based discrimination exacerbated this problem. Migration often resulted in difficulty accessing care in the country of residence, and many went back to their country of origin to receive treatment (for instance from the Netherlands back to Greece, and from Austria back to the UK).

Most participants had between three and five HCPs they needed to see on a regular basis, including gynaecologists, endocrinologists, surgeons, anaesthesiologists, pelvic floor therapists, mental health therapists, sexologists, nutritionists and acupuncturists. None reported having a unified team that would discuss a treatment plan in a multidisciplinary way, and therapies were extremely segmented and segregated from one another across all countries represented in the focus groups. Participants described frequently not reporting all relevant information during history taking and in follow-up appointments. Many used self-medication for managing the pain, such as CBD oil, and never told their HCPs. Withholding this type of information was reported by people living in countries with more restrictive attitudes and legislations relating to recreational drugs, such as Greece and Italy. Due to (the fear of) discrimination, misgendering and stigmatisation, participants had to constantly strike a balance between disclosing their gender identity and asking for affirmation or focusing on endometriosis treatment. Those who were using testosterone were anxious at the time testosterone was added to their medication list because all their HCPs would be able to see it.

“*It was kind of like: do I want to talk about my gender, or do I let it slip and get misgendered? (..) I decided not to speak up about it*.” (K., nonbinary person in their 20s from Australia)

## Discussion

### Summary main findings

This study explored the experiences of TGD people with endometriosis and, using an embodiment lens, examined the relationship between gender and endometriosis symptoms. It also assessed the accessibility and competence of the healthcare system in meeting the health needs of this marginalised community. Fourteen TGD individuals participated in online focus group discussions, organised into four groups. Each group met for two sessions with the same participants. The epistemological notions of embodiment allowed a comprehensive reflexive thematic analysis of the “emergent embodied phenotypes”^32^ of TGD people with endometriosis. The embodiment framework was helpful to appreciate how TGD people with endometriosis (1) understand gender and the menstrual cycle as reciprocal processes contingent upon their bodily existence, (2) contextualise cyclic pain as a multilevel gendered phenomenon integrating somatic, psychological and social contributing factors, (3) tell or withhold life stories with their bodies, and (4) struggle to access adequate endometriosis care due to the consequences of diverse forms of social inequalities.

### Experience of TGD people with endometriosis

TGD people experience endometriosis symptoms in idiosyncratic ways. For example, TGD people are frequently distressed by atypical symptoms such as chest/top tenderness that are considered “atypical” for cisgender women. Diagnostics and first-line treatment as recommended by current guidelines were often described as inadequate for this community.^1,25^ TGD people with endometriosis currently encounter great barriers to access endometriosis diagnosis and treatment.^12,13,16^ Some of these struggles overlap with those found in the pre-existing literature to affect cisgender women with endometriosis, such as medical gaslighting and fragmented healthcare systems.^40^ Others appear to be unique to this community, such as pervasive misgendering in gynaecological practices, since cisgender women do not experience misgendering. Coordinating GAC and endometriosis treatment hardly happens regardless of the setting and level of expertise of HCPs, which leaves patients with suboptimal therapeutic plans. Gender and menstrual cycle interplay for TGD people throughout their lives, evolving at different life stages depending on bodily processes as well as contextual factors. Menstruating and experiencing endometriosis-related pain were associated with gender dysphoria, dissociation and affective symptoms. For many TGD people, cycle suppression and higher testosterone levels relate to the feelings of gender affirmation and relief. TGD people with endometriosis often have tremendous preoccupations about their future, and worry about their priorities, goals and wishes in different life domains. Interestingly, subfertility was rarely reported as a major concern among TGD individuals with endometriosis, in contrast to previous studies on cisgender women, where fertility-related issues are often described as highly impactful.^41,42^ However, a few TGD participants who experienced infertility due to endometriosis did express grief over the loss of the ability to carry and have biologically related children. Overall, participants were more concerned about the impact of endometriosis on their careers and future employability, for instance, whether they would be able to complete their educational programmes or succeed in competitive fields such as academia.

### Implications for health policy and practice

Investigating gender as an embodied experience and understanding how this phenomenon interplays with endometriosis throughout one’s life course is helpful to overcome abled-male-body normativity in endometriosis discourse.^15^ Endometriosis typically impacts gendered processes such as menstruation, reproduction and penetrative sex. Gender roles and norms come with gendered expectations that, in the case of endometriosis, often cannot be fulfilled: for example, endometriosis-related subfertility impeding someone from becoming a “mother”.^12^ A more fluid and versatile conceptualisation of gender enables not only inclusivity of gender-diverse experiences but also less rigid gender norms and expectations for cisgender women. A profound sociocultural shift is, however, still needed to recognise and depathologise gender diversity and its implication in bodily experiences. Such a shift should result in an overall structural reframing of gender in healthcare systems to overcome the able male body norm and to meet TGD people’s health needs, mitigating health disparities which disproportionally affect marginalised communities. This study shows how structural barriers such as diagnostic delays caused by symptom dismissal, lack of HCPs’ knowledge, and gendered clinical environments, directly impede TGD people's access to timely and competent care. These barriers not only delay diagnosis but also force individuals to choose between disclosing their gender identity and receiving competent treatment.

Framing these challenges through the WHO’s rights-based availability, accessibility, acceptability, and quality framework makes it clear that such inequities constitute a violation of TGD individuals’ right to health.^29^ As suggested in previous studies, healthcare must be *available* through trained, competent providers; *accessible* without discrimination; *acceptable* in its recognition of gender diversity and bodily autonomy; and of high *quality*, integrating multidisciplinary and affirming approaches.^12,13,43^ The current pervasive lack of coordinated, gender-affirming care as highlighted by participants’ experiences demonstrates that these rights are not being met. Therefore, integrating TGD experiences into endometriosis care is not merely an inclusion effort but an urgent obligation of healthcare systems to uphold the human right to health, and advance health justice.

### Suggestions for future studies

Further studies in the field on endometriosis among TGD people are urgently needed.^44^ These are imperative not only to identify more adequate diagnostics and effective treatment for endometriosis for this underserved community but also because they could contribute to deeper understanding of how body phenomena and gender interplay overall.^12,13,16^ Critical theories such as gender, queer, and crip theories are essential to design and implement relevant research. Policy analysis should be conducted to efficiently identify structural barriers and possible windows of interventions. As previously described in ref.,^12^ participatory research methods and transdisciplinary approaches are needed for further research in the field of endometriosis and gender diversity to counteract the historical marginalisation of the TGD community.

### A note on researchers’ reflexivity

For many participants, it was the first time they had encountered other TGD people with endometriosis and many gave outstandingly positive feedback on their experience of participating in such discussions, as they found them validating and empowering. Some participants also said that they found the presence of a trans medical doctor on the team comforting and reassuring as well as unexpected, since they had only met cisgender HCPs so far. These trans connections are not only a research finding but an integral part of the dynamic process in which discussion facilitator and participants interact to create knowledge.^37^ Even more so, the nonbinary embodiment of many research team members allowed them to expand the idea of embodiment and embodied gender within and beyond the theoretical framework. The relevance and implications in leading a trans-focused research as someone who has the experience of being misgendered should not be overlooked.^37^ Without this experience, it would have been hard to grasp the liminality and the implications of the “fragile art of passing”^45^ or to understand how gender is not done simply by using GAC or changing pronouns; gender is an experience which rests under the skin. Being trans while doing trans research also implies that if on the one hand, there was a quicker, more self-evident trans connection and relatability between researchers and participants, on the other hand there were ways in which the facilitator was scrutinised and addressed in their own transness and masculinities. There are specific ways in which masculinities are done by and between transmasculine people, and the fact that many participants as well as the main facilitator were masculine leaning implied that these dynamics applied most of the time. Whereas the masculine subject in research remains largely overlooked, and even more so transmasculine subjects, a team reflection about masculinities within the group identified mostly patterns of subordinated and marginalised masculinities.^46,47^ We did not, however, encounter the risk identified by Robertson^47^ regarding masculine research subjects and their tendency to resist self-reflection and self-analysis. This may be due to the experience of misgendering more than the privileges associated with passing as masculine, or perhaps due to the extensive experience with self-reflection through years of feminine socialisation. This resistance, as Robertson^47^ suggests, may stem from an unconscious internalisation of masculine norms that view emotional introspection or spending time in self-reflection as inappropriate or “wrong”. We were questioned and challenged in our doing of gender, femininities, masculinities, and transness, which implied an additional necessary layer of reflexivity during and beyond the duration of the study.

### Strengths

The current study positions itself in a largely under-researched field. Its novelty lies not only in its objectives but also in the methodologies implemented. The wide outreach of the recruitment call guaranteed valuable heterogeneity in the participants’ background. Many geographical areas were represented, spanning from Canada, to Greece, and to Australia, and participants shared their inside perspectives of many different healthcare systems, allowing for a secondary comparative analysis which gave additional validation to our findings relating to the incompetence of healthcare systems for TGD people. The participants had various gender and life experiences as well as endometriosis stages. Juxtaposing such diverse experiences and yet finding the common thread between them contributed to creating a truly representative image of what the tapestry of TGD experiences with endometriosis looks like. Bringing together the experiences of TGD teenagers with those of middle-age postmenopausal TGD people – who had lived with endometriosis for over 30 years at the time of the study – greatly enriched the group discussion and led to indispensable insight. It was possible to thoroughly investigate the life course perspective thanks to the sharing of stories about the same topic from different life phases, for example, how late teenagers worry about their ability to finish their degrees and middle-aged people struggle to keep full-time jobs. The focus group design gave the unique opportunity to enhance participants’ responses because they responded to each other in a dialogical format while interacting with the facilitator.

### Limitations

Whereas gender identities were extremely varied, no participant identified as binarily transgender. Thus the voices and perspective of binary transmen remained underexplored in this study. The group was racially not sufficiently diverse, and more studies are needed to investigate the intersectionality of race in the current topic.^16^ Similarly, experiences from the Global North were overrepresented, and Global South perspectives remain unfortunately silenced in the discourse. The qualitative nature of the study comes with the restriction typical of these methodologies. It is possible that, given the researchers’ affiliation with the TGD community, participants may have felt pressured to respond in ways that aligned with the researchers’ perceived views or that were considered more socially desirable within the community. A quantitative approach mixed with the current research could have provided more quantifiable insights into the experiences of TGD people with endometriosis and their cycle, while expanding the external validity of the results. Emerging approaches such as the intersectional multilevel analysis of individual heterogeneity and discriminatory accuracy (I-MAIHDA) would be helpful in identifying health inequalities affecting TGD people at different intersections even when sample sizes are limited.^48^

## Conclusion

This study investigated the experiences of TGD people with endometriosis in relation to their cycle and gender, and identified barriers to access adequate diagnosis and treatment. Embodiment epistemological notions were a helpful tool to analyse and contextualise the stories that were told and withheld by TGD people with endometriosis. This community faces somehow similar, yet also profoundly distinct challenges, to cisgender women with endometriosis. Further research in this field is necessary to support a paradigm shift within and beyond the healthcare system to reorganise endometriosis care and GAC in competent and inclusive ways, and to ultimately mitigate healthcare disparities that disproportionately affect the TGD community.

## Author contributions

Conceptualisation: MG, JB, KJ, PV, AN. Methodology: MG, JB, KJ, PV, AN. Validation: MG, PV, AN. Formal analysis: MG, DR, PV. Investigation: MG, JB, KJ, DR. Resources: MG, JB, KJ, PV, AN. Software: JB, KJ. Data curation: MG, DR, PV, AN. Writing (original draft): MG, PV, AN. Writing (review and editing): MG, JB, KJ, DR, PV, AN. Visualisation: MG, AN. Supervision: MG, PV, AN. Project administration: MG, JB, KJ, AN. Funding acquisition: MG, AN.

## Provenance statement

This article was not commissioned and went through external peer review.
